# Evidence for an association of serum microanalytes and myofascial pain syndrome

**DOI:** 10.1186/s12891-023-06744-9

**Published:** 2023-08-01

**Authors:** Aishwarya Pradeep, Aybike Birerdinc, Travis Branigan, Vy Phan, Hailey Morris, Jay Shah, Secili DeStefano, Siddhartha Sikdar, John Srbely, Dinesh Kumbhare, Antonio Stecco, James Paik, Lynn H. Gerber

**Affiliations:** 1 Rehabilitation Medicine Department, Clinical Center, National Institutes of Health, 9000 Rockville Pike, , Bethesda, MD 20892 USA; 2 College of Science, George Mason University, 4400 University Drive, Fairfax, VA 22032 USA; 3 Department of Human Health and Nutritional Sciences, University of Guelph, 50 Stone Road East, Guelph, ON N1G 2W1 Canada; 4 College of Public Health, George Mason University, 4400 University Drive, Fairfax, VA 22030 USA; 5 Volgenau School, George Mason University, 4400 University Drive, Fairfax, VA 22032 USA; 6 Department of Medicine, Division of Physical Medicine and Rehabilitation, University of Toronto, 200 Elizabeth Street, Toronto, ON M5G 2C4 Canada; 7 Department of Physical Medicine and Rehabilitation, New York University Langone Medical Center, 550 First Avenue, New York, NY 10016 USA; 8 Medicine Service Line, Inova Health System, 3300 Gallows Rd, Falls Church, VA 22042 USA

**Keywords:** Myofascial Pain Syndrome, Serum Microanalytes, Cytokines

## Abstract

**Background:**

Myofascial Pain Syndrome (MPS) is a common pain disorder. Diagnostic criteria include physical findings which are often unreliable or not universally accepted. A precise biosignature may improve diagnosis and treatment effectiveness. The purpose of this study was to assess whether microanalytic assays significantly correlate with characteristic clinical findings in people with MPS.

**Methods:**

This descriptive, prospective study included 38 participants (25 women) with greater than 3 months of myofascial pain in the upper trapezius. Assessments were performed at a university laboratory. The main outcome measures were the Beighton Index, shoulder range of motion, strength asymmetries and microanalytes: DHEA, Kynurenine, VEGF, interleukins (IL-1b, IL-2, IL-4, IL-5, IL-7, IL-8, IL-13), growth factors (IGF-1, IGF2, G-CSF, GM-CSF), MCP-1, MIP-1b, BDNF, Dopamine, Noradrenaline, NPY, and Acetylcholine. Mann–Whitney test and Spearman’s multivariate correlation were applied for all variables. The Spearman’s analysis results were used to generate a standard correlation matrix and heat map matrix.

**Results:**

Mean age of participants was 32 years (20–61). Eight (21%) had widespread pain (Widespread Pain Index ≥ 7). Thirteen (34%) had MPS for 1–3 years, 14 (37%) 3–10 years, and 11 (29%) for > 10 years. The following showed strong correlations: IL1b,2,4,5,7,8; GM-CSF and IL 2,4,5,7; between DHEA and BDNF and between BDNF and Kynurenine, NPY and acetylcholine. The heat map analysis demonstrated strong correlations between the Beighton Index and IL 5,7, GM-CSF, DHEA. Asymmetries of shoulder and cervical spine motion and strength associated with select microanalytes.

**Conclusion:**

Cytokine levels significantly correlate with selected clinical assessments. This indirectly suggests possible biological relevance for understanding MPS. Correlations among some cytokine clusters; and DHEA, BDNF kynurenine, NPY, and acetylcholine may act together in MPS. These findings should be further investigated for confirmation that link these microanalytes with select clinical findings in people with MPS.

## Background

Myofascial pain syndrome (MPS) is a common and often chronic pain syndrome that affects an estimated 15% of people seeking medical attention in the US [1]. However, because of lack of agreement among clinicians and investigators about criteria for diagnosis, this number may not accurately reflect the prevalence. MPS is a non-articular musculoskeletal pain condition that has posed significant challenges to individuals worldwide by interfering with daily routines [2]. It is a syndrome that can be acute or chronic, whose distribution can be local, regional or referred and may be distressing [3–6]. There are additional descriptors for MPS as well, though these may not be universally accepted. Descriptions of the findings associated with MPS have changed over the years [7], and diagnostic criteria as proposed by Travell and Simons [8, 9], while frequently used, have not been universally accepted. One component frequently used for the diagnosis of MPS is the presence of myofascial trigger points (MTrPs). These can be active (spontaneously painful) or latent (painful only upon compression). The pain associated with compression of latent MTrPs or with movement is qualitatively similar to the pain associated with active MTrPs [10].

MPS is also accompanied by several other debilitating symptoms and findings, including but not limited to a restricted range of motion in the region of pain. This is commonly seen with cervical spine and/or shoulder motion. Moreover, psychological distress (including depressive symptoms and anxiety) and fatigue accompany MPS, negatively impacting activities of daily living (walking, bathing, etc.), and disturbing sleep [11]. Other chronic conditions that share similar clinical features or occur as comorbidities include fibromyalgia and sickness behaviors associated with chronic fatigue syndrome [12–14]. Potential mechanisms for precipitating and perpetuating chronic pain such as neuroinflammatory responses, hyperalgesic priming, and central sensitization have been known to serve as common processes in all of these conditions. [15, 16]. Due to the heterogeneity in the cluster of symptoms associated with MPS and overlap with other chronic conditions across individuals, MPS has been difficult to diagnose using objective, standardized measures in the clinic and in research. Furthermore, the underlying pathophysiology of MPS has also not been fully elucidated, creating a need for additional investigation in this field. As a result, it has been challenging to provide effective, long-lasting treatments for those suffering from these conditions.

One of the key outstanding challenges in the field includes the lack of consensus on the diagnostic criteria for MPS and what is meant by chronic MPS [3]. A review of the literature has documented that the majority of publications referencing MPS in fact include trigger points and taut band of muscle as part of the diagnostic criteria [17, 18]. However, finding trigger points accurately is dependent upon palpation, and the reliability of palpation has been challenged by some [17–19]. These deficiencies should be acknowledged and may be improved by identifying and adopting additional metrics that may improve reliability.

It is important to have reliable, objective clinical criteria for establishing medical syndromes. This is not only necessary for diagnosis and prognosis but for the creation and delivery of low-risk, minimally invasive and optimal treatment plans given the current opioid crisis in the United States. To address knowledge gaps in understanding MPS, it would be beneficial to expand the biosignature to include objective measures, such as biochemical analytes, and physical and possibly cognitive performance measures. As such, this study aims to determine if there is a reliable correlation between microanalytic profiles in the serum and selected clinical measures frequently associated with MPS.

## Methods

This study was approved by George Mason University Institutional Review Board. It was conducted according to the Code of Ethics of the World Medical Association (Declaration of Helsinki) for research involving humans. All participants provided informed written consent prior to participating.

We recruited research participants through patient referrals and flyers posted throughout our community seeking people with chronic MPS. Inclusion criteria were based upon self-reports and physical findings from musculoskeletal assessments [8, 9]. The requirement for inclusion was a 3 month history of myofascial pain. There were no comparison groups to serve as controls (i.e. did not have pain). The presence of this was determined by an existing diagnosis of MPS or from the self-reported history provided by the research participant. MPS is defined as a non-articular musculoskeletal pain disorder often accompanied by a hyperirritable nodule associated with spontaneous (unprovoked) or induced pain. Characteristics of this pain include deep aching of muscle and fascia with possible limitation of motion and/or muscle weakness. Referred pain is possible. Exclusion criteria included history of cervical spine or shoulder girdle fracture, presence of cervical radiculopathy, diagnosis of neurodegenerative disease or stroke or recent surgery (6 months).

The physical examination assessments were performed exclusively by experienced clinicians whose inter-rater reliability for physical assessment including MTrP palpation was shown to be high from earlier work [11]. The evaluation included soft-tissue palpation of the trapezii for 1) a taut band, and hyperirritable nodules and 2) identification of referred pain and a twitch response, if present. Palpation was routinely made at four designated sites a reported in a prior publication [11]: 1 cm medial to the left and right acromioclavicular joint; and 2 cm lateral to the spinous process of C8 on the left and right. A Kendall 10-point manual muscle test [1] and active assisted range of motion (ROM) were performed for shoulder flexion, abduction, internal/external rotation and cervical flexion/extension, side bending and rotation and degree of asymmetry in range of motion was calculated by subtracting the measured ROM from the normal on the left and right sides. A ratio of the differences between the two was calculated and provided %asymmetry. A score of tissue extensibility was determined using the Brighton Criteria and Beighton score. A pressure algometer (Commander Algometer, Tech Medical, Salt Lake City, UT; http://www.jtechmedical.com/Commander/commander-algometer) was used to measure the degree of local tenderness and reported as a pressure pain threshold (PPT) as measured in pounds/square inch. Higher scores mean a higher pain threshold. Pressure algometry was performed bilaterally on all subjects. Algometry procedures had been previously determined to be valid and reliable [20]. Patient-reported outcomes included a verbal rating scale (VAS) for pain intensity (ranging from 0–10) and widespread pain index (WPI) [21]. The procedures followed have been previously published [11].

A fasting morning blood draw was performed on all participants. The analytes studied included dehydroepiandrosterone (DHEA), Kynurenine, vascular endothelial growth factor (VEGF), interleukins (IL-1b, IL-2, IL-4, IL-5, IL-7, IL-8, IL-13), insulin-like growth factors (IGF-1, IGF-2), granulocyte colony-stimulating factor (G-CSF), granulocyte–macrophage colony-stimulating factor (GM-CSF), monocyte chemoattractant protein-1/monocyte chemotactic and activating factor (MCP-1/MCAF), macrophage inflammatory protein-1beta (MIP-1b), brain-derived neurotrophic factor (BDNF), Dopamine, Noradrenaline, neuropeptide Y (NPY), and Acetylcholine. The pro-and anti-inflammatory cytokines and the IGF panel were profiled using a Bio-Plex 200 Array System allowing simultaneous identification and quantification of multiple different analytes in a single biomolecular assay (Bio-Rad). This system functions by measuring proteins bound to the surfaces of fluorescent microspheres providing highly accurate, real-time digital analysis of serum samples as small as 12.5 μl. The remaining analytes were measured using standard enzyme-linked immunoassay (ELISA) assays (Promega, Abcam, Abnova). All ELISA assays were run on freshly thawed and aliquoted serum samples according to each manufacturer’s protocols. Additionally, all ELISA assays were run with standard curves and were performed in triplicates.

Our research team composed a statistical analysis plan prior to data collection, and it is described as follows. A distribution and boxplot analysis of the data was performed to demonstrate the biological diversity in the cohort. A pair-wise non-parametric analysis, specifically, a Mann–Whitney test, was performed for all variables between the widespread pain and the non-widespread pain cohort (*p* = 0.05). A Spearman’s multivariate correlation was performed on all of the variables in question with a *p*-value cut-off of 0.05. In addition, the analytes were grouped into biologically relevant sectors and a heat map matrix was generated based on the *p*-values of the correlation table to identify areas of signal intensity based on the Spearman’s analysis results.

## Results

Thirty-eight participants (25 women) met the criteria for chronic myofascial pain syndrome of the shoulder/trapezius region lasting more than 3 months. The mean age of participants was 32 years (range 20-61 years). Eight of 38 (21%) had widespread pain as determined by a score on the WPI ≥ 7 [19]. Thirteen of 38 (34%) had MPS duration of 1–3 years, 14 (37%) had duration of 3–10 years, and 11 participants (29%) had MPS for more than 10 years. Medication usage included dietary supplements, reported by 10 participants, and included vitamins to help manage pain, stress, sleep, headache and fatigue. Eight participants took no medication, 10 took nonsteroidal anti-inflammatory drugs (NSAIDs) routinely for pain and mobility, 5 took antidepressants and 5 took gabapentin. Only one patient routinely took an opiate (tramadol). Most participants used non-pharmacological treatments for pain relief including massage, heat, ice, stretch, strengthening exercise, yoga, physical therapy and dry needling. Massage was employed by 22 participants.

Clinical descriptions of pain location and intensity are presented in Table 1. Table 2 presents frequency and temporal patterns of the pain. Table 3 presents the distribution of MTrPs and their status (active, latent or neither). Table 4 presents the mean pain level, as determined by PPT for each type of myofascial trigger point.

**Table 1:** Pain symptom characteristics for patients (*n* = 38)

Type of Pain:	Dull	Aching	Burning	Tight/Stiff	Sharp/Stabbing	Hot
(n)	17	14	3	9	8	10

**Table 2:** Nature of pain

Pain Pattern	Inconsistent	Intermittent	Constant	Morning	Evening	No Pattern
(n)	16	15	23	9	12	17

**Table 3:** Active trigger point location^a^

Site of Active Trigger Point	Number of Trigger Points	Trigger Points at Site/All Trigger Points	Percentage of Trigger Points at Specific Site
Site 1	1	1/36	2.78%
Site 2	14	14/36	38.89%
Site 3	20	20/36	55.56%
Site 4	1	1/36	2.78%

^a^Site 1 = Medial acromioclavicular region on left; Site 2 = Lateral to spinous process of C7 in upper trapezius on left; Site 3 = Lateral to spinous process of C7 in upper trapezius on right; Site 4 = Medial acromioclavicular region on right

**Table 4:** Distribution of mean pain pressure threshold measures relative to myofascial trigger point and widespread pain

Types of Trigger Point	Number of Sites Represented (*n* = 156)	Mean Pain Pressure Threshold Measurement (ppsi^a^)
Active Trigger Point	32	6.9 (.6–20)
Latent Trigger Point	31	9.3 (2.8–20)
No Trigger Point	92	11.8 (3.8–20)
Widespread Pain	N/A	4.35 (2.9–7.2)

^a^*ppsi* pounds/square inch

Table 5 displays additional clinical details from the cohort including a self-reported level of pain Brief Pain Inventory (BPI), WPI and VAS. Self-reports of additional related symptoms, fatigue, headache and difficulty thinking were assessed. Participants with widespread pain had higher pain scores than those without (p = ns). Participants with widespread pain reported significantly greater symptom severity than those with scores < 7 on WPI, the cutoff for widespread pain (*p* = 0.005). The mean Beighton Score was 2.9 (range 0–8) and not hyper-extensible. However, there were 12/35 (35%) measured in the cohort with a score > 4, which is considered hyper-extensible.

**Table 5:** Mean symptom measurements of pain, headache, and fatigue

**Worst Pain, BPI (Range: 1–12)**	4.5
Average Pain, BPI (Range: 1–12)	2.9
Main Pain Score, WPI (Range: 1–20)	10.4
Pain Measure, VAS (Range: 1–10)	2.96
Fatigue Score (Range: 0–3)	0.57
Headache Score (Range: 0–3)	1.5
Trouble Thinking (Range: 0–3)	0.7

*BPI* Brief Pain Inventory, *WPI* Widespread Pain Inventory, *VAS* Verbal Analogue Scale

We selected 2 different analytic approaches to the data in order to determine whether there might be a meaningful association, suggestive of relationships worth exploring using larger sample sizes.

**Fig. 1:**
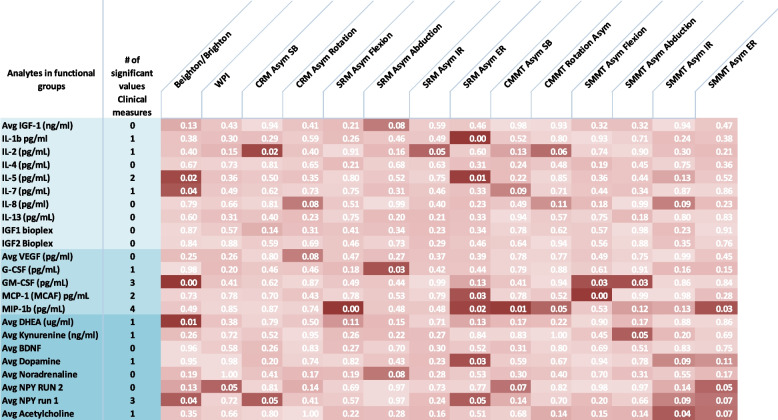
Heat Map of Correlations between Microanalytes and Clinical Measures. Legend: Rows: specific analytes assayed. They are categorized into 3 groups (cytokines, growth factors and neuropeptides/catecholamines) based on their biological similarities. Columns: specific clinical measures that had been included in the clinical evaluations. Abbreviations: CRM = cervical range of motion; Asym = asymmetry; SB = sidebending; SRM = shoulder range of motion; IR = internal rotation; ER = external rotation; CMMT = cervical manual muscle testing; SMMT = shoulder manual muscle testing. Statistically significant associations are in the darkest red for ease of visualization

Figure 1 presents the first approach using a heat map, with groupings of variables based on likely biological similarities that have correlations with the serum analytes that may merit further exploration. Both asymmetrical range of motion and manual muscle testing are often associated with pain, as reported in prior studies [2, 10]. The findings identify significant associations between measures of asymmetries (range of motion and manual muscle testing) and cytokines and myokines. Additionally, the measure selected for joint laxity (the Beighton index) is associated with a cytokine associated with collagen disorders (IL-5) and by 2 myokines. The former suggests a biological link between laxity and this cytokine. This lends some biological support for the finding.

**Fig. 2:**
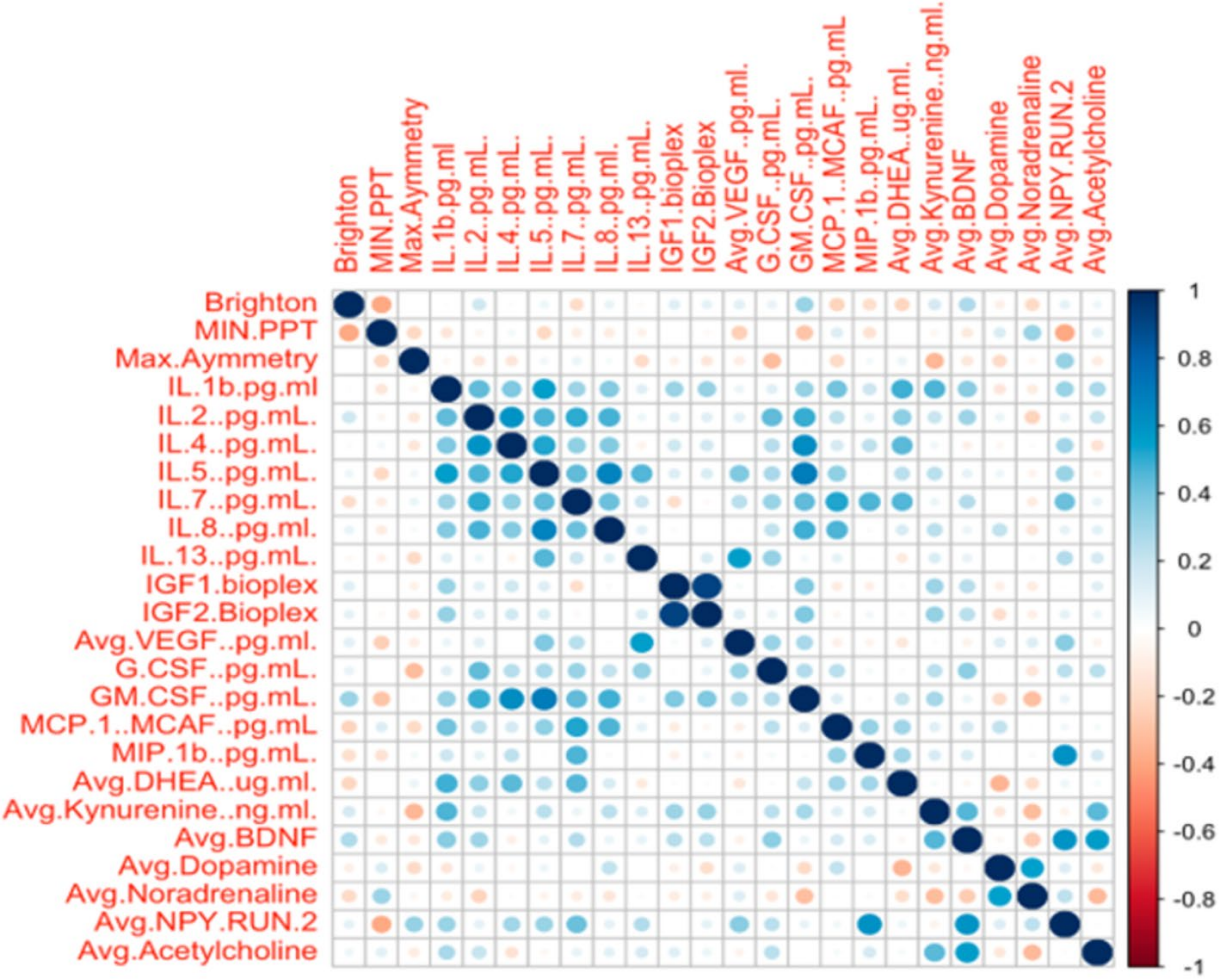
Correlation Matrix. Legend: Magnitude of the correlation is displayed in terms of color from negative to positive correlation (Y axis on right) and size of circle

The second approach was the standard correlation matrix with the microanalytes that were assessed (Fig. 2), which presents correlations among the cytokines. The correlations involving GM-CSF include many of these cytokines, as well. BDNF correlated with NPY and acetylcholine.

## Discussion

There is concern that diagnostic criteria for MPS are imprecise and that this poses difficulties in selecting evaluations and treatments that have general acceptance [5–7]. Some investigators suggest that MTrPs should be a requirement for diagnosis [22]. Others believe that MPS is a musculoskeletal pain syndrome with contributions of fascia and underlying nerve contributions and that the diagnosis does not require the presence of a hyperirritable nodule [23]. These conflicting views encourage investigators to explore more quantitative and precise instrumentation for the examination of patients and agreement on which tissues need to be assayed for this precision.

As of this writing, no firm consensus exists about which measures are necessary for assuring uniformity of diagnosis and evaluating treatment effectiveness, trends do exist for each. In a publication from our group [6] that examined MPS publications, five clusters were commonly selected to describe the syndrome: “trigger points,” “muscle,” “pain,” “nervous system,” and “fascia.” These seem like reasonable domains to accept in the effort to standardize contributors to MPS and hence should be included in clinical evaluations, when feasible. The presence of a taut band and hypersensitive spot during clinical examination are often cited as a necessary condition for the diagnosis, with patterns of referred pain and/or ability to elicit a twitch response as being possibly but not necessarily required for diagnosis [22]. These evaluations are clinical, often routine, and rarely require expensive equipment or take an inordinate amount of time in a busy clinic. However, other measures, such as those described in this paper, namely serological measures of growth factors and cytokines are yet to be proven to have clinical value and may not be easily adaptable to routine laboratory assays.

Research opportunities exist as well and can possibly provide quantitative measures that may be more sensitive and specific for diagnosing MPS. These may lead to a better understanding of mechanisms for MPS and help distinguish acute from chronic pain. There are biochemical data [24–28] to support the concept that pro-inflammatory cytokines, neuropeptides and catecholamines may be contributing to the pain syndrome, but further validation is needed especially to assess whether samples need be taken from tissue, peripheral blood and/or cerebrospinal fluid. Importantly, the analytes need to be linked to a meaningful clinical finding and/or biological process to provide usefulness.

In our view, self-report of pain is a necessary condition for MPS. Additionally, objective data can provide important information about its intensity and impact on organ systems (e.g., the neuromusculoskeletal system), thus improving diagnostic accuracy and possibly helping to elucidate underlying pain mechanisms. There is value in linking reliable and quantitative clinical observations with pain reports, but as already mentioned, the assays need to be practical and affordable.

The roles of different biochemical analytes (interleukins, growth factors, neuropeptides etc.) as biosignatures have been examined in pain syndromes and may have utility as endpoints for clinical trials [15, 29]. Some have reported elevations of various pro-inflammatory cytokines (i.e., IL-6, 8,12), tumor necrosis factor, monocyte chemoattractant protein-1 and growth factors. We have confirmed some of these findings and expanded observations to expand the discussion to explore biologically relevant relationships associated with clinical findings, such as linkage between connective tissue laxity and pain. There is still a lack of consensus in the field as to whether they can be linked to clinical symptoms and function to provide meaningful information for diagnosis and possible treatment outcome evaluation. Additionally, while the utility of each analyte as a biomarker has been studied in an individual context, this study aims to investigate the relationships among classes of analytes that share similar functions, with the goal of assessing their correlations with clinical measures. Both objective measures and self-reports, we believe, are essential for diagnosing MPS, assessing severity and for determining treatment outcomes.

To accommodate the small sample size and the high number of variables, we adopted two strategies. We selected clinical variables commonly assessed in practice that are efficient and inexpensive. These include reliable measures of pain, standardized self-reports, and objective measures frequently associated with pain conditions, such as range of motion, tissue extensibility, sensory testing and measures of PPT. We analyzed these measures in terms of correlations with various biochemical analytes. We grouped these according to their classification as inflammatory cytokines, growth factors or neuropeptides/catecholamines in order to assess whether these might be indicative of biological processes and biochemical pathways suggestive of mechanisms for pain generation and control. For example, the clustering of IL-5 and IL-7 with elevated Beighton scores is aligned with reports about IL-5 associating with stiffness following joint replacement and possible collagen abnormalities [30].

The analytical approach used in this paper chose two different methods (i.e., the correlation matrix and heat map) to try to identify possible meaningful correlations between important clinical findings in people with MPS. We selected physical findings that we routinely perform and are likely to be performed in most clinics (e.g., range of motion and pain measures, such as PPT, VAS and WPI) in an effort to link these with microanalytes.

The interpretation of these data is, at best, preliminary and dependent upon corroboration from other studies and larger sample sizes. The small sample size and absence of a control for comparison (i.e. a no-pain group), are limitations of the study. Nonetheless, the data analytic approach is of interest because of the relationships found among various analytes and clinical findings. The Beighton/Brighton score significantly correlates with IL-5, IL-7 and GM-CSF, DHEA and NPY1. Asymmetries in range of motion (shoulder and neck), which we have previously reported to be associated with pain levels and active MTrPs, have associations with various cytokines, indirectly suggesting these microanalytes may accompany chronic musculoskeletal pain [11].

We suggest that this preliminary report provides data that are worthy of continued exploration. It is suggestive of possible biologically relevant relationships among various analytes and select clinical findings that are frequently present in people with MPS. Further, investigating these microanalytes may be a first step in identifying a biochemical signature and possible pathways to explain chronic MPS.

## Conclusions

Asymmetries in range of motion (shoulder and neck), which we have previously reported to be associated with pain levels and active MTrPs, have associations with various cytokines, indirectly suggesting these microanalytes may associate with chronic musculoskeletal pain. We also identified correlations among microanalytes (cytokine clusters IL-1,2,4,5, and 7,8) and DHEA, BDNF kynurenine, NPY and acetylcholine), suggesting that these molecules may act in concert and/or synergistically. This preliminary report provides some data worthy of continued exploration. It is suggestive of possible biologically relevant relationships among various analytes and select clinical findings that are frequently present in people with MPS. Further, investigating these microanalytes may be a first step in identifying a biochemical signature and possible pathways to explain and target treatments for chronic MPS.

## Data Availability

The datasets used and/or analyzed during the current study are available from the corresponding author on reasonable request.

## Abbreviations

MPS: Myofascial pain syndrome
MTrP: Myofascial trigger point
ROM: Range of motion
PPT: Pressure pain threshold
VAS: Verbal rating scale
WPI: Widespread pain index
DHEA: Dehydroepiandrosterone
VEGF: Vascular endothelial growth factor
IL: Interleukin
IGF: Insulin-like growth factors
G-CSF: Granulocyte colony-stimulating factor
GM-CSF: Granulocyte–macrophage colony-stimulating factor
MCP-1/MCAF: Monocyte chemoattractant protein-1/monocyte chemotactic and activating factor
MIP-1b: Macrophage inflammatory protein-1beta
BDNF: Brain-derived neurotrophic factor
NPY: Neuropeptide Y
NSAID: Nonsteroidal anti-inflammatory drug
BPI: Brief pain inventory
ELISA: Enzyme-linked immunoassay
